# APOEε4 Carriers Exhibit Objective Cognitive Deficits: A Cross-Sectional Study in a Single Center Trial

**DOI:** 10.3390/brainsci14030281

**Published:** 2024-03-15

**Authors:** Yanfang Zeng, Wenying Du, Mingkai Zhang, Ariel Walker, Ying Han, Yuchuan Ding

**Affiliations:** 1 Department of Neurology, Xuanwu Hospital of Capital Medical University, No. 45 Changchun Street, Xicheng District, Beijing 100053, China; zyf2075697@163.com (Y.Z.); zhangmingk0527@163.com (M.Z.); 2 Department of Neurology, Beijing Luhe Hospital of Capital Medical University, Beijing 101100, China; 3 Department of Neurology, China Japan Friendship Hospital, Beijing 100029, China; duwyneurology@163.com; 4 Department of Neurological Surgery, Wayne State University School of Medicine, 550 E Canfield, Detroit, MI 48201, USA; ariel.walker@med.wayne.edu (A.W.); yding@med.wayne.edu (Y.D.); 5 School of Biomedical Engineering, Hainan University, Haikou 570228, China; 6 Center of Alzheimer’s Disease, Beijing Institute for Brain Disorders, Beijing 100053, China; 7 National Clinical Research Center for Geriatric Diseases, Beijing 100053, China; 8 Institute of Biomedical Engineering, Shenzhen Bay Laboratory, Shenzhen 518132, China

**Keywords:** apolipoprotein E, cognitive function, mild cognitive impairment, Alzheimer’s disease, plasma amyloid-β

## Abstract

Objective: To explore the association between the apolipoprotein E (APOE) genotype and objectively assessed cognitive function. Methods: In this cross-sectional study, 537 participants underwent a neuropsychological assessment for cognitive function and blood testing for APOE genotype. Based on cognitive test results, participants were stratified into two cohorts: Cognitively Unimpaired participants (CU) and Cognitively Impaired participants (CI). The CI group was further divided into Mild Cognitive Impairment (MCI) and Alzheimer’s Disease (AD). Furthermore, we conducted age stratification, categorizing participants into three age groups: age 1: <65 years, age 2: 65–75 years, and age 3: >75 years. We assessed the disparities in cognitive function associated with ε4 carrier status across different age brackets. Plasma amyloid-β levels were measured in a cohort of 294 participants to investigate potential interactions involving ε4 carrier status, diagnosis, sex, or plasma markers. Results: The APOE genotypic distribution among the 537 participants was characterized as follows: ε2/ε2 (5 participants), ε2/ε3 (67), ε2/ε4 (13), ε3/ε3 (330), ε3/ε4 (113), and ε4/ε4 (9). Allele frequencies were: ε3 at 78.21%, ε4 at 13.41%, and ε2 at 8.38%. Notably, the ε4 carrier frequency was markedly elevated in the AD group at 81.8% when compared to MCI at 32.8% and CU at 21.3% (*p* < 0.05). Within the Cognitively Unimpaired (CU) cohort, the sole discernible contrast between ε4+ and ε4− emerged in STT-B (*p* < 0.05). Within the CI group, ε4 carriers showed statistically poorer scores as compared to non-ε4 carriers in several cognitive tests (*p* < 0.05). Age stratification result revealed that, among ε4 carriers, cognitive function scores within the age 3 group were significantly inferior to those of age 1 and age 2 groups (*p* < 0.05). Plasma amyloid-β detection was applied to the 294 participants. We tested plasma amyloid-β (Aβ42) and plasma amyloid-β (Aβ40) levels and calculated the Aβ42/Aβ40 ratio. We found that among female ε4 carriers, both Aβ42 and the Aβ42/Aβ40 ratio were notably lower than their male counterparts (*p* < 0.05). Conclusions: The ε3/ε3 was the most prevalent among participants, succeeded by ε3/ε4 and ε2/ε3. The least prevalent were ε2/ε4, ε4/ε4, and ε2/ε2 genotypes. The ε3 was predominant, followed by the ε4 and ε2. Individuals with the ε4 allele exhibited significant cognitive impairment, with an especially high prevalence in AD group at 81.8%. The study unveils a pronounced correlation between the ε4 allele and cognitive deficits, implying its potential role in the advancement and severity of cognitive disorders, notably Alzheimer’s disease. Cognitive function declines with age in individuals carrying the ε4, and women are more affected by ε4.

## 1. Introduction

Alzheimer’s disease (AD) is a neurodegenerative disorder typified by memory impairment and cognitive deterioration. It represents the prevailing form of dementia among the elderly [1,2]. There is currently no cure to the disease [3], and it typically is accompanied by some mental symptoms [4,5,6,7,8,9]. Around 13% of the global population aged 65 and above is afflicted with Alzheimer’s disease (AD), with the prevalence escalating to an alarming 45% among individuals aged 85 and older. On a worldwide scale, it is prognosticated that the prevalence of Alzheimer’s disease will escalate to encompass 80 million individuals by the year 2050, which poses a great burden on the global society and family economy [10,11,12].

In accordance with the age of disease onset, Alzheimer’s disease (AD) can be dichotomized into early onset AD (EOAD) and late-onset AD (LOAD). EOAD, recognized as familial AD, manifests as an autosomal dominant disorder with onset occurring prior to the age of 65, constituting approximately 5% of all AD cases [13,14]. The majority of EOAD instances are attributed to mutations in the amyloid precursor protein (APP), presenilin 1 (PSEN1), or presenilin 2 (PSEN2) genes [15]. Conversely, LOAD, denoted as sporadic AD, represents the predominant category in AD cases. In the exploration of numerous genetic susceptibility loci associated with LOAD, the apolipoprotein E (APOE) gene stands out as the most robust risk factor [16]. The APOEε4 allele is acknowledged as a prominent risk factor and the most potent genetic contributor to AD [17,18], particularly in the context of its late-onset manifestation [19].

Mild cognitive impairment (MCI), specifically the amnestic subtype recognized as the prodromal stage of Alzheimer’s disease (AD) [20], signifies the transitional phase between the typical aging process and the onset of dementia. MCI is characterized by cognitive decline beyond what is anticipated based on an individual’s age and educational background, without significant impairment in daily activities and lacking evidence of dementia. The prevalence in population-based epidemiological studies among adults aged 65 and older ranges from 3% to 19%. Viewing mild cognitive impairment as a precursor to dementia underscores its potential significance, as early recognition may facilitate the implementation of secondary prevention strategies.

APOEε4 is a risk factor for AD characterized primarily by cognitive impairment. Current studies on AD, MCI, and the APOEε4 allele indicate a correlation between APOEε4 and compromised cognitive function among MCI and AD patients. The presence of APOEε4 also predicted an increased conversion rate of MCI to AD [21,22]. However, conclusions about the role of APOEε4 in normal cognitive functioning people were inconsistent. Some studies found APOEε4 did not have a negative effect on older adults with normal cognitive function [23,24], while other studies had shown that ε4 carriers had poorer cognitive function compared to ε4 non-carriers [25]. Among the myriad factors influencing the impact of APOEε4, age emerges as a paramount determinant. The deleterious effect of APOEε4 on human cognitive function exhibits a correlation with age, with the detrimental influence intensifying as age advances [26]. Amyloid-β (Aβ) is the characteristic pathological manifestation of AD, whether plasma amloid-β (Aβ) in APOEε4 populations can determine AD disease status needs further study [27]. There were limited recruitment-based investigations on APOE genotyping in well-characterized Chinese cohorts, and those that existed were conducted on a small scale [28,29].

We conducted a large-sample, cross-sectional study of northern Chinese people in a Single Center Trial to investigate the distribution of APOEε4 in the Chinese population. Elucidating its involvement in Alzheimer’s disease (AD) patients is imperative for substantiating the association between APOEε4 and compromised cognitive function in individuals with Mild Cognitive Impairment (MCI) and Alzheimer’s Disease (AD). Our goal was to evaluate the influence of APOE ε4 on normal cognitive function and pinpoint the stage at which its effects became more prominent, indicating the initial signs of the AD. We scrutinize the interplay among APOEε4, age, diagnosis, sex, and plasma markers to ascertain the varying degrees of risk impact across diverse populations. Lastly, our aim was to explore the potential correlation between the degree of cognitive dysfunction and the ratio of ε4, and investigating whether APOEε4 plays a determining role in the severity of cognitive impairment. The overarching goal was to achieve swifter detection of Alzheimer’s disease within the population predisposed to AD.

## 2. Participants and Methods

### 2.1. Participants

From August 2016 to October 2022, 537 participants were recruited by advertisements at Xuanwu Hospital, Capital Medical University (Sino Longitudinal Study on Cognitive Decline of National Clinical Medical Research Center for Geriatric Diseases) [30]. Inclusion criteria for enrollment were 50–79 years old with an education level ≥ 9 years and Mandarin-speaking, all participants have provided written informed consent either individually or through their legal guardians/families. They received tests for global cognitive, language, and executive function. All participants underwent comprehensive testing, including assessments for APOE genotype and plasma Aβ index. which were integrated with cognitive assessment results. MCI and AD were thus confirmed.

The exclusion criteria encompass the following: 1. Neurological disorders that may lead to a decline in cognitive abilities (e.g., Parkinson’s disease, encephalitis, brain tumors, stroke or epilepsy). 2. History of psychiatric illness. 3. Congenital intellectual developmental delay. 4. Other diseases that may contribute to a decline in cognitive abilities (e.g., severe anemia, thyroid dysfunction, syphilis, or HIV). 5. Severe depression and anxiety. 6. Traumatic brain injury resulting in cognitive impairment.

MCI and AD Participants were considered as Cognitively Impaired participants (CI). The normal cognitive function participants were defined as Cognitively Unimpaired participants (CU). The CU (Cognitively Unimpaired) group consisted of individuals categorized as normal controls (NC) and those exhibiting symptoms of Subjective Cognitive Decline (SCD). To explore the association with age, a cohort of 537 participants was stratified into three age groups: age 1 (<65 years), age 2 (65–75 years), and age 3 (>75 years). Plasma tests were conducted on only 294 participants to examine potential interactions related to ε4 carrier status, diagnosis, sex, or plasma markers.

### 2.2. NC, SCD, MCI and AD Inclusion Criteria

NC: 1. The neuropsychological scale exhibited normalcy; however, it did not satisfy the diagnostic criteria for Mild Cognitive Impairment (MCI) and dementia. 2. Participants failing to fulfill the diagnostic criteria for Subjective Cognitive Decline (SCD) among cognitively unimpaired individuals.

SCD [30]: 1. Sustained cognitive deterioration in self-perception relative to previously established normative cognitive states; 2. Performance within the normal range on objective cognitive assessments, equated for age, gender, and educational background; Fails to meet criteria for mild cognitive impairment or dementia; 3. Apprehensions linked to self-reported cognitive decline grievances.

MCI [30]: Satisfy any of the following three criteria while not meeting the criteria for dementia: 1. Attainment of impaired scores (defined as >1 standard deviation below the age-corrected normative means) on both measures within at least one cognitive domain (memory, language, or speed/executive function); 2. Display of impaired scores in each of the three cognitive domains assessed (memory, language, or speed/executive function); 3. Achievement of a score ≥ 9 on the Functional Activities Questionnaire (FAQ).

AD [31,32,33]: 1. Satisfy the diagnostic criteria indicative of dementia; 2. The onset of the disease is insidious, and the symptoms appear gradually in months to years; 3. Have a clear history of cognitive impairment; 4. It is manifested as amnesic syndrome (decline in learning and near memory with impairment of one or more other cognitive domains) or non-amnesic syndrome (impairment of language, visuospatial, or executive function with impairment of one or more other cognitive domains). 5. CDR score ≥1. 6. Rule out other causes of dementia.

### 2.3. Neuropsychological Assessments

Participants underwent several neuropsychological assessments for cognitive function, including global cognitive function (MMSE, MoCA-B, MES), memory function (AVLT-D, AVLT-R), language function (VFT, BNT), and executive function (STT-A, STT-B), described as follows. 1. Chinese version of Mini-Mental State Examination (MMSE) [34]: Normal range: illiterate: ≥17, education level 1–6 years: ≥20, education level >6 years: ≥24. 2. The Montreal Cognitive Assessment Basic (MoCA-B) [35]: Normal range: illiterate and primary school: ≥19, Junior middle school: ≥22, college: ≥24. 3. Auditory Verbal Learning Test-long delayed memory (AVLT-D), Auditory Verbal Learning Test- recognition (AVLT-R) [36]: Normal range: AVLT-D: 50–59 years old: ≥5, 60–69 years old: ≥4, 70–79 years old: ≥3; AVLT-R 50–59 years old: ≥20, 60–69 years old: ≥19, 70–79 years old: ≥18. 4. Shape Trail Test Parts A and B (STT-A) (STT-B) [37]: Normal range: STT-A, 50–59 years old: ≤70 s, 60–69 years old: ≤80 s, 70–79 years old: ≤100 s; STT-B, 50–59 years old: ≤180 s, 60–69 years old: ≤200 s, 70–79 years old: ≤240 s. 5. Verbal Fluency Test (VFT) [38]: Normal range: Junior middle school: ≥12, high school: ≥13, college: ≥14. 6. Boston Naming Test (BNT) [39]: Normal range: Junior middle school: ≥20, high school: ≥21, college: ≥22. 7. Memory And Executive Screening (MES) [40]: Normal range: ≥75 points is abnormal.

### 2.4. Blood Tests for APOE Genotype

The APOE genotyping will be conducted through the standard Sanger sequencing method utilizing the following primers: 5′-ACGCGGGCACGGCTGTCCAAGG-3′ (forward) and 5′-GGCGCTCGCGGATGGCGCTGA-3′ (reverse). The APOE amplification conditions are as follows: one cycle at 98 °C for 10 s, 35 cycles at 72 °C for 5 s, and one cycle at 72 °C for 5 min. The polymerase chain reaction (PCR) will be performed in a final volume of 30 μL, using PrimeStar HSDNA polymerase (Takara Bio, Kusatsu, Shiga, Japan) with GC buffer. The reaction will include 10 pmol of both forward and reverse primers, as well as 50 ng of genomic DNA template.

### 2.5. Plasma Amyloid-β

Plasma Aβ concentrations will be quantified utilizing a commercially accessible kit, namely the V-PLEX Aβ Peptide Panel 1 (6E10) Kit (K15200E) from Mesoscale Diagnostics, located in Rockville, Maryland, USA. Duplicate measurements of Aβ peptide levels will be conducted for each blood draw, utilizing identical aliquots.

### 2.6. Statistical Analysis

The outcomes were subjected to statistical analysis employing the SPSS 20.0 software package. Presentation of data was expressed as the mean ± standard deviation. Quantitative data underwent comparison through either the *t*-test, whereas categorical data underwent comparison utilizing the χ^2^ test. A generalized linear model was employed to scrutinize the interplay between APOE4 carriage status and either age or diagnosis concerning cognitive performance. Additionally, the model explored the interaction between APOE4 carriage status and gender in relation to plasma amyloid protein levels.

## 3. Results

### 3.1. Participant Demographics and Genetic Makeup

In our investigation of 537 individuals, we discovered a diverse range of APOE genotypes among these participants. The most prevalent genotype was ε3/ε3, found in a majority of participants. This was followed by the ε3/ε4 and ε2/ε3 genotypes. Genotypes ε2/ε4, ε4/ε4, and ε2/ε2 were notably less common (Table 1). When we looked at allele frequencies, the ε3 allele was predominant in our participant pool, while the ε4 and ε2 alleles were less frequent.

### 3.2. Cognitive Performance across Different Groups

Our study categorized participants into two main groups: those who were cognitively unimpaired (CU) and those who exhibited cognitive impairment (CI). The CI group was further divided into patients with Mild Cognitive Impairment (MCI) and those with Alzheimer’s Disease (AD) (Table 2). A striking observation emerged when we examined the frequency of the ε4 among these groups. In the AD group, the presence of the ε4 was remarkably high, standing at 81.8%. This was significantly more prevalent than in the MCI group, where it was found in 32.8% of individuals, and in the CU group, where only 21.3% carried this allele.

### 3.3. Cognitive Test Outcomes

When it came to the cognitive assessments, we noticed some intriguing patterns. In the CU group, the only significant difference between those carrying the ε4 allele and those who did not was observed in the Shape Trail Test Part B (STT-B). ε4 carriers in this group demonstrated poorer scores, suggesting a unique pattern of cognitive performance (Table 3). However, the scenario was quite different in the CI group. Here, ε4 carriers exhibited poorer scores across a range of cognitive tests, including the Mini-Mental State Examination (MMSE), Montreal Cognitive Assessment-Basic (MoCA-B), Auditory Verbal Learning Test-Delay (AVLT-D), Auditory Verbal Learning Test-Recognition (AVLT-R), Verbal Fluency Test (VFT), and Memory Encoding Switch (MES) (Table 4). These results were statistically significant, underscoring the influence of the ε4 allele on cognitive performance. Interestingly, ε4 carriers also scored poorer in the Shape Trail Test Part A (STT-A), a pattern consistent with the findings in the CU group.

### 3.4. A Results of Age Interaction Analysis

**Figure 1:**
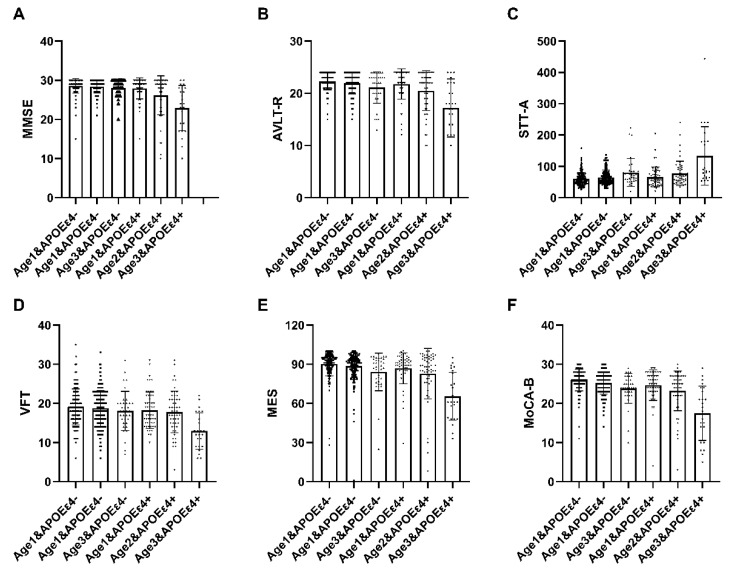
The interactive analysis of age and ε4 carrier status on cognitive function. In individuals with APOEε4+, statistically significant differences were observed in MMSE, MoCA-B, MES, AVLT-R, VFT, and STT-A among the three groups, demonstrating a progressive decline with advancing age. Abbreviation: MMSE, Chinese version of Mini-Mental State Examination; MOCA-B, The Montreal Cognitive Assessment Basic; AVLT-D, Auditory Verbal Learning Test-long delayed memory; AVLT-R, Auditory Verbal Learning Test-recognition; STT-A, Shape Trail Test Parts A; STT-B, Shape Trail Test Parts B; VFT, Verbal Fluency Test; BNT, Boston naming test; MES, Memory And Executive Screening. APOEε4+, ε4 carriers, APOEε4−, ε4 non-carriers. age 1: <65 years, age 2: 65–75 years, age 3: >75 years.

A total of 537 participants underwent age interaction analysis. Within the APOEε4+ subgroup, statistically significant distinctions were observed in comprehensive cognitive function (MMSE, MoCA-B, MES), memory function (AVLT-R), language function (VFT), and executive function (STT-A) across the three delineated groups (*p* < 0.05) (Table 5). These findings suggest that cognitive impairment tends to intensify with advancing age, and this phenomenon is notably accentuated in individuals carrying the ε4 allele. The interaction between cognitive function and age among ε4 carriers was further explored utilizing a general linear model (Figure 1).

### 3.5. Analysis of Diagnosis and Plasma Marker Interaction

**Figure 2:**
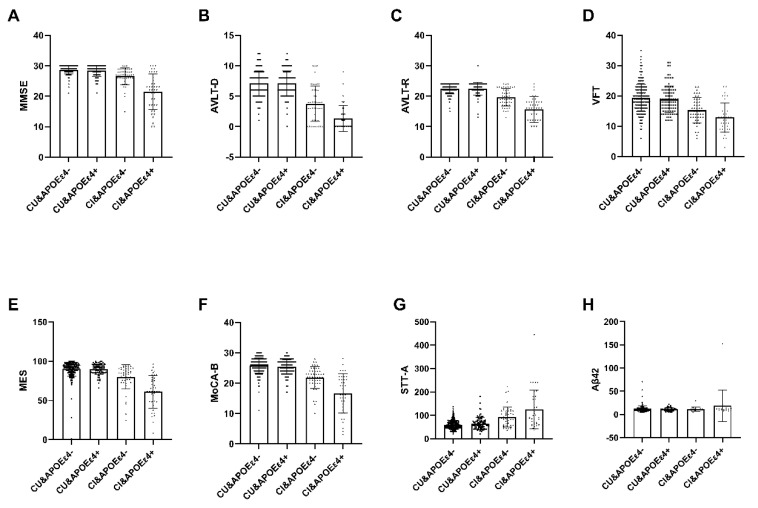
The interactive analysis between ε4 carrier status, diagnosis and plasma marker. Within the Cognitive Impaired (CI) group, notable distinctions emerged between the APOEε4+ group and the APOEε4− group in various cognitive assessments, namely MMSE, MoCA-B, MES AVLT, VFT, STT-A, and Plasma amyloid-β (Aβ42) levels (*p* < 0.05). Conversely, within the Cognitive Unimpaired (CU) group, no significant disparities were observed. Abbreviation: MMSE, Chinese version of Mini-Mental State Examination; AVLT-D, Auditory Verbal Learning Test-long delayed memory; AVLT-R, Auditory Verbal Learning Test- recognition; VFT, Verbal Fluency Test; MES, Memory And Executive Screening; MoCA-B, The Montreal Cognitive Assessment Basic; STT-A, Shape Trail Test Parts A; CU, Cognitive unimpaired; CI, Cognitive impairment; APOEε4+, ε4 carriers, APOEε4−, ε4 non-carriers. Aβ42, Plasma amyloid-β42.

Among the 537 recruited subjects, only 294 participants classified as APOEε4+ (*n* = 71) or APOEε4− (*n* = 223) completed the blood sample analysis, which encompassed measurements of Aβ42 and Aβ40 (Table 6). Within the Cognitive Impaired (CI) group, notable distinctions emerged between the APOEε4+ group and the APOEε4− group in various cognitive assessments, namely MMSE, MoCA-B, MES AVLT, VFT, STT-A, and Plasma amyloid-β (Aβ42) levels (*p* < 0.05). Conversely, within the Cognitive Unimpaired (CU) group, no significant disparities were observed (Figure 2).

### 3.6. Analysis of the APOE Interaction by Gender

**Figure 3:**
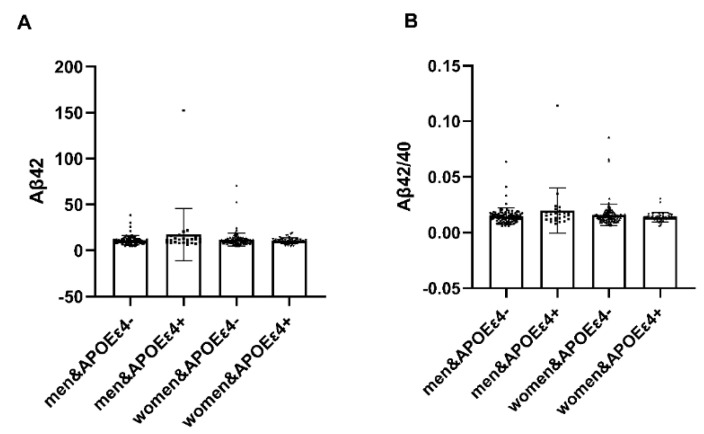
Analysis of APOE interaction by gender. In individuals with APOEε4+, notable distinctions emerged between males and females regarding plasma amyloid-β (Aβ42) and the Aβ42/Aβ40 ratio (*p* < 0.05). Plasma amyloid-β (Aβ42) and the Aβ42/Aβ40 ratio in females were lower. Conversely, within APOEε4−, no significant disparities were observed. Abbreviation: Aβ42, Plasma amyloid-β42; Aβ40, Plasma amyloid-β40; APOEε4+, ε4 carriers, APOEε4−, ε4 non-carrier.

In individuals with APOEε4+, notable distinctions emerged between males and females regarding plasma amyloid-β (Aβ42) and the Aβ42/Aβ40 ratio (*p* < 0.05). Females exhibited heightened vulnerability to the deleterious impact of APOEε4, while in AP-OEε4− individuals, no significant differences were observed (Table 7). It illustrates the interaction effect between APOE4 carriage status and gender on plasma amyloid protein levels (Figure 3).

## 4. Discussion

Our study highlighted the predominance of the ε3/ε3 genotype among participants, subsequently trailed by ε3/ε4 and ε2/ε3. The high prevalence of the ε4 allele, especially in the AD group, points to its significant association with cognitive deficits. This correlation is particularly pronounced in the context of Alzheimer’s disease, suggesting the potential role of the ε4 allele in the progression and severity of cognitive disorders. This correlation is particularly prominent in the realm of Alzheimer’s disease, indicating the potential impact of the ε4 allele on the advancement and severity of cognitive disorders.

The human apolipoprotein E (ApoE) gene is located on sub-band 2 of band 13 of the long arm of chromosome 19 (19q13.2) [41]. ApoE is a polymorphic 299-amino acid protein with a molecular weight of 34,200. ApoE gene has three alleles (ε2, ε3 and ε4) coding for three protein isoforms: APOEε2, APOEε3 and APOEε4. These isoforms exhibit distinctions at residues 112 and 158. APOEε3 features Cys-112 and Arg-158, APOEε4 showcases arginine at both positions, while APOEε2 manifests cysteine at both loci [42].

APOE has 6 genotypes (ε2/ε2, ε3/ε3, ε4/ε4, ε2/ε3, ε2/ε4, ε3/ε4), which constitute genetic polymorphism. The frequency of APOE genotype was shown to vary in different populations, but it was highest with ε3/ε3, followed by ε2/ε3 and ε3/ε4, and lowest with ε2/ε2, ε2/ε4 and ε4/ε4 [43,44]. Our findings illustrated that the frequency of the ε3/ε3 homozygous genotype was the most prominent (330 cases), succeeded by the frequencies of the ε3/ε4 and ε2/ε3 genotypes (113 and 67 cases, respectively). The frequencies of the ε2/ε4, ε4/ε4, and ε2/ε2 genotypes were the least observed (13, 9, and 5 cases, respectively). The ε2, ε3 and ε4 gene frequencies in our restricted study were 8.38%, 78.21%, and 13.41%, respectively, the ε2, ε3 and ε4 gene frequencies in previous studies were 5%~10%, 70%~80%, 10%~15% in distinct human populations. Our research results are basically consistent with other research results [42,45].

The ε4 carrier (ε4+) frequency was considerably higher in the AD group (81.8%) when compared to MCI (32.8%) and CU (21.3%) (*p* < 0.05). We can find that the more severe the cognitive impairment, the higher the ratio of ε4.

Of the 9 cognitive tests, only the score of Shape Trail Test Parts B (STT-B) was poorer in ε4 carriers than in non-ε4 carriers in the CU group (*p* < 0.05). No statistically significant distinctions were discerned in the remaining 8 cognitive function assessments. In brief, there was no discernible variance in cognitive function between individuals who carried the ε4 allele and those who did not within the CU group. APOEε4 stands out as the most potent genetic risk factor for Alzheimer’s disease (AD); nevertheless, the impact of APOEε4 on cognition in cognitively healthy adults remains a subject of contention in prior research [46]. Our research found no influence of APOEε4 on cognition in CU adults. We hope that our results can provide a theoretical basis for future research.

In the CI group, although ε4 carriers implied no significant differences in the Shape Trail Test Parts B (STT-B) and Boston naming test (BNT), ε4 carriers showed statistically poorer scores compared to non-ε4 carriers in several cognitive assessments, including: MMSE, MoCA-B, AVLT-D, AVLT-R, VFT, MES and STT-A. All these variances were significant. These results suggested that APOEε4 carriers implied poorer cognitive performance. APOEε4 accelerated cognitive decline in MCI and AD participants compared to non-carriers. This is coincident with previous studies. This suggests that APOEε4 has a role in accelerating progression of cognitive function impairment [47,48].

In the context of APOEε4’s role in Alzheimer’s disease (AD), numerous factors have been investigated, including age, gender, plasma markers, and others; however, diverse conclusions have been drawn. Our study on the correlation between these factors and APOEε4 has yielded corresponding findings. Among the various factors influencing the impact of APOEε4, age emerges as one of the most significant determinants. The deleterious effect of APOEε4 on human cognitive function is closely linked to age. With increasing age, the negative impact of APOEε4 becomes more pronounced. Furthermore, women exhibit heightened susceptibility to the adverse effects of ε4.

APOE polymorphic alleles represent the principal genetic determinants of Alzheimer’s disease [11], with the presence of APOEε4 being associated with heightened susceptibility to the onset of the condition. In contrast to the ε3/ε3 genotype, each instance of the ε4 allele amplifies the risk of Alzheimer’s disease (AD) by approximately threefold, while the presence of two copies escalates the risk to 8–14 times that of individuals with the ε3/ε3 genotype. The potential mechanism lies in the impact of APOEε4 on the synthesis and elimination processes of amyloid-β (Aβ) [49,50,51,52]. The findings of Barthel with co-authors similarly substantiate this assertion [53]. Conversely, the APOEε2 allele confers a protective influence. The Alzheimer’s Disease (AD) risk among ε2 allele carriers is merely 0.6 of that observed in ε3/ε3 genotype carriers [54,55,56]. In a comprehensive exploration of cognitive domains encompassing verbal memory, working memory, executive function, and verbal ability, Wolk et al. observed diminished verbal memory in ε4 carriers relative to non-carriers among AD patients displaying compromised working memory, executive function, and verbal ability [56]. Their results were consistent with the present study.

Numerous investigations involving individuals with Mild Cognitive Impairment (MCI) have consistently reported a diminution in global cognition and memory performance associated with the presence of the APOEε4 allele. This signifies a correlation between the APOEε4 allele and compromised memory function in both middle-aged and elderly subjects diagnosed with MCI. Moreover, the APOEε4 genotype serves as a predictive indicator for an accelerated decline in overall cognition within the MCI cohort. Notably, individuals carrying the ε4 allele exhibited a significantly more rapid cognitive decline, coupled with an elevated conversion rate to Alzheimer’s Disease (AD) [22,57,58].

Our study reveals a notable correlation that a higher prevalence of the ε4 allele is associated with a greater degree of cognitive impairment. This finding aligns with other reports providing mechanistic insights into the pathogenicity of the ε4 allele. The presence of APOEε4, as demonstrated in our research, seems to hold significant diagnostic value in identifying Alzheimer’s Disease (AD) within dementia-prone populations.

Amyloid-β (Aβ) stands as the distinctive pathological hallmark of Alzheimer’s disease (AD), and ongoing research is dedicated to exploring plasma biomarkers associated with the pathology of this neurodegenerative condition. Presently, the detection of plasma amyloid-β (Aβ) proves instrumental in identifying the characteristic pathology of AD, thereby significantly enhancing the diagnostic efficacy for AD’s distinctive pathological features [27]. Our affirmative findings pertaining to analyses involving Aβ42 and the Aβ42/Aβ40 ratio in APOEε4+ women, suggest the active involvement of ε4 in the pathological alterations associated with AD. These results, in turn, offer a more precise biochemical representation of the Alzheimer’s disease risk.

Despite these findings, we acknowledge a primary limitation of our study: its design as a single-center, cross-sectional investigation conducted on a homogenous population. This limitation notwithstanding, the association of APOEε4 with varying degrees of cognitive impairment across all stages of AD is noteworthy. It underscores the potential utility of APOEε4 testing as a tool for early identification of patients at an increased risk of developing dementia. Such early detection could lead to heightened clinical vigilance and potentially more effective management strategies in the early stages of the disease.

These findings provide valuable insights into the genetic factors that may influence cognitive health and the progression of cognitive impairments, particularly Alzheimer’s disease. They underline the importance of considering genetic makeup when assessing cognitive function and the potential for targeted interventions in populations with different APOE genotypes. To fully understand the implications of incorporating APOEε4 testing into clinical practice, further investigation is warranted. Larger, longitudinal studies involving more diverse populations would be invaluable. Such studies would provide a more comprehensive assessment of the risks and benefits associated with APOEε4 testing, potentially leading to more nuanced and effective clinical strategies for the management and early detection of Alzheimer’s disease.

## 5. Conclusions

In summary, our results demonstrated that the ε3/ε3 was the most prevalent among participants, succeeded by ε3/ε4, ε2/ε3, ε2/ε4, ε4/ε4, and ε2/ε2 genotypes. The ε3 was predominant, followed by the ε4 and ε2. Individuals with the ε4 allele exhibited significant cognitive impairment, with an especially high prevalence in AD group. The study unveils a pronounced correlation between the ε4 allele and cognitive deficits, implying its potential role in the advancement and severity of cognitive disorders, notably with increasing age, the negative impact of APOEε4 becomes more pronounced. Furthermore, women exhibit heightened susceptibility to the adverse effects of ε4. This could lead to more nuanced and effective clinical strategies for the management and early detection of Alzheimer’s disease.

## Figures and Tables

**Table 1:** APOE Genotyping Result and Allele Frequency.

Genotype						Allele Frequency		
ε2/ε2	ε2/ε3	ε2/ε4	ε3/ε3	ε3/ε4	ε4/ε4	ε2	ε3	ε4
5	67	13	330	113	9	90 (8.38%)	840 (78.21%)	144 (13.41%)

**Table 2:** Comparison of Number of Cases with or without ε4 Allele in Different Groups.

	AD	MCI	CU	χ²	*p*
ε4(+) (n%)	18 (81.8%)	21 (32.8%)	96 (21.3%)	43.114	<0.001 ***
ε4(−) (n%)	4 (18.2%)	43 (67.2%)	355 (78.7%)		
Total	22	64	452		

*** *p* < 0.001.

**Table 3:** The Score of Cognitive Function with Different APOE Genotypes in Cognitive Unimpaired Group.

	ε4(+) (*n* = 96)	ε4(−) (*n* = 355)	Χ^2^/t	*p*
Sex (men/women)	30/66	124/231	0.455	0.545
Age (years)	65.60 ± 6.27	65.15 ± 6.32	−0.623	0.534
Education level	12.89 ± 2.84	12.81 ± 3.06	−0.230	0.818
MMSE	28.33 ± 1.80	28.70 ± 1.42	1.841	0.068
MoCA-B	25.32 ± 2.46	25.88 ± 2.51	1.944	0.053
AVLT-D	7.08 ± 2.11	7.07 ± 2.09	−0.054	0.957
AVLT-R	22.44 ± 2.11	22.34 ± 1.59	−0.504	0.614
STT-A	65.31 ± 25.37	60.05 ± 19.63	−1.884	0.062
STT-B	152.0 ± 48.19	139.8 ± 45.52	−2.229	0.027 *
VFT	18.84 ± 4.45	19.35 ± 4.48	0.983	0.326
BNT	25.02 ± 2.81	24.91 ± 2.97	−0.329	0.743
MES	89.73 ± 6.53	90.17 ± 8.93	0.454	0.650

* *p* < 0.05. Abbreviation: MMSE, Chinese version of Mini-Mental State Examination; MoCA-B, The Montreal Cognitive Assessment Basic; AVLT-D, Auditory Verbal Learning Test-long delayed memory; AVLT-R, Auditory Verbal Learning Test- recognition; STT-A, Shape Trail Test Parts A; STT-B, Shape Trail Test Parts B; VFT, Verbal Fluency Test; BNT, Boston naming test; MES, Memory And Executive Screening.

**Table 4:** The Score of Cognitive Function with Different APOE Genotypes in Cognitive Impaired Group (MCI and AD).

	ε4(+) (*n* = 39)	ε4(−) (*n* = 47)	χ²/t	*p*
Sex (men/women)	12/27	28/19	7.109	0.008 **
Age (years)	70.97 ± 8.38	68.95 ± 6.81	−1.258	0.212
Education level	12.69 ± 2.87	11.72 ± 3.35	−1.425	0.158
MMSE	21.41 ± 5.84	26.57 ± 2.80	5.063	<0.001 ***
MoCA-B	16.62 ± 6.39	21.89 ± 3.66	4.571	<0.001 ***
AVLT-D	1.36 ± 2.13	3.74 ± 2.90	4.387	<0.001 ***
AVLT-R	15.69 ± 4.29	19.60 ± 2.92	4.999	<0.001 ***
STT-A	125.18 ± 82.41	93.64 ± 42.65	−2.162	0.035 *
STT-B	253.72 ± 107.47	221.04 ± 108.46	−1.397	0.166
VFT	12.90 ± 4.78	15.23 ± 4.14	2.428	0.017 *
BNT	20.77 ± 5.33	21.94 ± 5.01	1.044	0.299
MES	61.04 ± 20.78	79.85 ± 15.42	0.034	<0.001 ***

*** *p* < 0.001 ** *p* < 0.01 * *p* < 0.05. Abbreviation: Cognitive impaired group included MCI and AD; MCI, Mild cognitive impaired; AD, Alzheimer’s disease; MMSE, Chinese version of Mini-Mental State Examination; MoCA-B, The Montreal Cognitive Assessment Basic; AVLT-D, Auditory Verbal Learning Test-long delayed memory; AVLT-R, Auditory Verbal Learning Test-recognition; STT-A, Shape Trail Test Parts A; STT-B, Shape Trail Test Parts B; VFT, Verbal Fluency Test; BNT, Boston naming test; MES, Memory And Executive Screening.

**Table 5:** The interactive examination of cognitive function in relation to both age and ε4 carriage status.

	ε4− Age 1 (*n* = 177)	ε4− Age 2 (*n* = 195)	ε4− Age 3 (*n* = 30)	ε4+ Age 1 (*n* = 56)	ε4+ Age 2 (*n* = 59)	ε4+ Age 3 (*n* = 20)	B	*p*
MMSE	28.59 ± 1.84	28.39 ± 1.62	28.08 ± 2.13	27.95 ± 2.70	26.18 ± 4.98	22.92 ± 5.77	1.504	<0.001 ***
MoCA-B	25.98 ± 2.78	25.19 ± 2.81	23.83 ± 3.81	24.66 ± 3.91	23.24 ± 5.09	17.50 ± 6.95	1.434	0.001 **
AVLT-D	7.18 ± 2.12	6.40 ± 2.53	5.75 ± 2.96	6.70 ± 2.45	5.44 ± 3.49	2.46 ± 3.06	0.598	0.055
AVLT-R	22.32 ± 1.63	21.91 ± 2.03	21.11 ± 2.99	21.89 ± 3.08	20.49 ± 3.81	17.21 ± 5.5	0.982	0.002 **
STT-A	59.59 ± 21.76	64.89 ± 23.20	39.12 ± 44.26	65.71 ± 31.84	77.51 ± 39.12	133.71 ± 93.28	−15.681	<0.001 ***
STT-B	134.74 ± 47.93	154.57 ± 56.47	193.31 ± 111.09	151.06 ± 51.89	176.0 ± 69.88	264.58 ± 117.09	−13.796	0.093
VFT	19.12 ± 4.59	18.78 ± 4.59	18.08 ± 5.01	18.25 ± 4.66	17.80 ± 5.35	12.96 ± 4.60	1.351	0.031 *
BNT	24.86 ± 2.80	24.60 ± 3.62	22.89 ± 4.43	24.70 ± 3.27	24.40 ± 3.34	20.29 ± 5.82	0.634	0.159
MES	90.29 ± 9.10	88.65 ± 10.46	84.11 ± 14.40	86.91 ± 11.81	82.82 ± 19.29	65.52 ± 18.24	4.197	0.006 **

*** *p* < 0.001 ** *p* < 0.01 * *p* < 0.05. Abbreviation: MMSE, Chinese version of Mini-Mental State Examination; MoCA-B, The Montreal Cognitive Assessment Basic; AVLT-D, Auditory Verbal Learning Test-long delayed memory; AVLT-R, Auditory Verbal Learning Test- recognition; STT-A, Shape Trail Test Parts A; STT-B, Shape Trail Test Parts B; VFT, Verbal Fluency Test; BNT, Boston naming test; MES, Memory And Executive Screening.

**Table 6:** The interactive analysis of cognitive function and plasma markers in relation to both diagnosis and APOE ε4 carriage status.

	ε4− CU (*n* = 201)	ε4− CI (*n* = 22)	ε4+ CU (*n* = 53)	ε4+ CI (*n* = 18)	B	*p*
MMSE	28.70 ± 1.42	26.57 ± 2.80	28.33 ± 1.80	21.41 ± 5.84	4.900	<0.001 ***
MoCA-B	25.88 ± 2.51	21.89 ± 3.66	25.32 ± 2.46	16.62 ± 6.39	4.950	<0.001 ***
AVLT-D	7.07 ± 2.09	3.74 ± 2.90	7.08 ± 2.11	1.36 ± 2.13	2.564	<0.001 ***
AVLT-R	22.34 ± 1.59	19.60 ± 2.92	22.44 ± 2.11	15.69 ± 4.29	4.149	<0.001 ***
STT-A	60.05 ± 19.63	93.64 ± 42.65	65.31 ± 25.37	125.18 ± 82.41	−26.247	<0.001 ***
STT-B	139.81 ± 45.52	221.04 ± 108.46	152.02 ± 48.19	253.72 ± 107.47	−21.588	0.119
VFT	19.35 ± 4.48	15.23 ± 4.14	18.84 ± 4.45	12.90 ± 4.78	2.182	0.039 *
BNT	24.91 ± 2.97	21.94 ± 5.01	25.02 ± 2.81	20.77 ± 5.33	1.109	0.155
MES	90.17 ± 8.93	79.85 ± 15.42	89.73 ± 6.53	61.04 ± 20.78	18.510	<0.001 ***
Aβ42	11.56 ± 6.50	11.51 ± 4.87	10.86 ± 3.63	18.72 ± 33.51	−7.521	0.036 *
Aβ40	765.34 ± 156.79	804.61 ± 164.09	725.30 ± 150.42	850.42 ± 166.30	−64.556	0.209
Aβ42/Aβ40	0.0155 ± 0.0091	0.0146 ± 0.0066	0.0148 ± 0.0053	0.0188 ± 0.0240	−0.005	0.190

*** *p* < 0.001 * *p* < 0.05. Abbreviation: MMSE, Chinese version of Mini-Mental State Examina tion; MoCA-B, The Montreal Cognitive Assessment Basic; AVLT-D, Auditory Verbal Learning Test-long delayed memory; AVLT-R, Auditory Verbal Learning Test- recognition; STT-A, Shape Trail Test Parts A; STT-B, Shape Trail Test Parts B; VFT, Verbal Fluency Test; BNT, Boston naming test; MES, Memory And Executive Screening. Aβ42, Plasma amyloid-β42; Aβ40, Plasma amyloid-β40; CU, Cognitive unimpaired; CI, Cognitive Impairment.

**Table 7:** The interactive analysis of cognitive function and plasma markers in relation to both gender and APOE4 carriage status.

	ε4− Men (*n* = 90)	ε4− Women (*n* = 133)	ε4+ Men (*n* = 25)	ε4+ Women (*n* = 46)	B	*p*
MMSE	28.14 ± 2.07	28.64 ± 1.54	26.10 ± 5.05	26.44 ± 4.52	0.018	0.972
MOCAB	24.88 ± 3.32	25.74 ± 2.67	22.05 ± 6.58	23.15 ± 5.13	−0.546	0.394
AVLT-D	6.01 ± 2.60	7.09 ± 2.25	4.98 ± 3.43	5.63 ± 3.31	0.266	0.562
AVLT-R	21.39 ± 2.18	22.40 ± 1.78	19.88 ± 4.89	20.76 ± 3.88	0.047	0.923
STT-A	67.19 ± 32.01	62.03 ± 20.99	84.80 ± 51.69	81.61 ± 57.93	0.142	0.982
STT-B	161.84 ± 77.77	141.69 ± 48.96	189.13 ± 77.68	177.91 ± 86.94	−4.970	0.676
VFT	18.66 ± 4.74	18.99 ± 4.56	16.33 ± 5.17	17.48 ± 5.32	−1.303	0.157
BNT	25.22 ± 3.41	24.16 ± 3.34	24.26 ± 4.49	23.58 ± 4.02	−0.725	0.272
MES	88.13 ± 11.04	89.48 ± 10.02	80.21 ± 18.90	81.99 ± 17.61	−1.139	0.616
Aβ42	11.16 ± 4.93	11.82 ± 7.16	17.31 ± 28.43	10.43 ± 3.16	7.662	0.007 **
Aβ40	768.52 ± 169.80	769.65 ± 149.15	765.43 ± 167.28	750.48 ± 161.41	15.577	0.704
Aβ42/Aβ40	0.0148 ± 0.0074	0.0158 ± 0.0097	0.0198 ± 0.0205	0.0136 ± 0.0041	0.007	0.015 *

** *p* < 0.01 * *p* < 0.05. Abbreviation: MMSE, Chinese version of Mini-Mental State Examination; MoCA-B, The Montreal Cognitive Assessment Basic; AVLT-D, Auditory Verbal Learning Test-long delayed memory; AVLT-R, Auditory Verbal Learning Test-recognition; STT-A, Shape Trail Test Parts A; STT-B, Shape Trail Test Parts B; VFT, Verbal Fluency Test; BNT, Boston naming test; MES, Memory And Executive Screening. Aβ42, Plasma amyloid-β42; Aβ40, Plasma amyloid-β40.

## Data Availability

The data presented in this study are available on request from the corresponding author. The data are not publicly available due to privacy.
